# Spatial variation in the management and outcomes of acute coronary syndrome

**DOI:** 10.1186/1471-2261-5-21

**Published:** 2005-07-11

**Authors:** Alain Vanasse, Théophile Niyonsenga, Josiane Courteau, Jean-Pierre Grégoire, Abbas Hemiari, Julie Loslier, Goze Bénié

**Affiliations:** 1 Family Medicine Department, Faculty of Medicine, *Université de Sherbrooke*, 3001, 12^th ^Avenue North, Sherbrooke (QC), Canada, J1H 5N4; 2 PRIMUS Group, Clinical Research Center, Sherbrooke University Hospital, Sherbrooke (QC), Canada; 3 Population Health Research Unit and Faculty of Pharmacy, *Université Laval*, Québec (QC), Canada; 4 Geography and Remote Sensing Department, *Université de Sherbrooke*, Sherbrooke (QC), Canada

## Abstract

**Background:**

Regional disparities in medical care and outcomes with patients suffering from an acute coronary syndrome (ACS) have been reported and raise the need to a better understanding of links between treatment, care and outcomes. Little is known about the relationship and its spatial variability between invasive cardiac procedure (ICP), hospital death (HD), length of stay (LoS) and early hospital readmission (EHR). The objectives were to describe and compare the regional rates of ICP, HD, EHR, and the average LoS after an ACS in 2000 in the province of Quebec. We also assessed whether there was a relationship between ICP and HD, LoS, and EHR, and if the relationships varied spatially.

**Methods:**

Using secondary data from a provincial hospital register, a population-based retrospective cohort of 24,544 patients hospitalized in Quebec (Canada) for an ACS in 2000 was built. ACS was defined as myocardial infarction (ICD-9: 410) or unstable angina (ICD-9: 411). ICP was defined as the presence of angiography, angioplasty or aortocoronary bypass (CCA: 480–483, 489), HD as all death cause at index hospitalization, LoS as the number of days between admission and discharge from the index hospitalization, and EHR as hospital readmission for a coronary heart disease ≤30 days after discharge from hospital. The EHR was evaluated on survivors at discharge.

**Results:**

ICP rate was 43.7% varying from 29.4% to 51.6% according to regions. HD rate was 6.9% (range: 3.3–8.2%), average LoS was 11.5 days (range: 7.5–14.4; median LoS: 8 days) and EHR rate was 8.3% (range: 4.7–14.2%). ICP was positively associated with LoS and negatively with HD and EHR; the relationship between ICP and LoS varied spatially. An increased distance to a specialized cardiology center was associated with a decreased likelihood of ICP, a decrease in LoS, but an increased likelihood of EHR.

**Conclusion:**

The main results of this study are the regional variability of the outcomes even after accounting for age, gender, ICP and distance to a cardiology center; the significant relationships between ICP and HD, LoS and EHR, and the spatial variability in the relationships between ICP and LoS.

## Background

Cardiovascular events represent a major health burden for modern societies and the acute coronary syndrome (ACS), defined as myocardial infarction or unstable angina, accounts for a large percentage of them [1]. Practice guidelines regarding ACS management have been widely published [2-4]. However, gaps have been observed between practice guidelines and how ACS is actually managed. Moreover, regional variations have been observed in the ACS management and outcomes [5-9]. This situation raises the need to better understand the link between treatment, care and outcomes in patients suffering from an ACS as well as to determine whether or not these relationships varied spatially. This article focuses on the link between invasive cardiac procedure (ICP), hospital death (HD), hospital length of stay (LoS) and early hospital readmission (EHR).

ICP requires technical facilities and a professional expertise available only in specialized cardiology centers. For patients suffering from an ACS, exposure to ICP during the first hospitalization for myocardial infarction is considered to be protective of a readmission for cardiac reasons [10]. What is less known is the effect ICP may have on HD, LoS and EHR, and how this effect may vary from one region to another.

Although the geographical localization of specialized centers for stroke treatment in Canada [11] has been described in terms of theoretical accessibility to specialized care for a specific population, a more comprehensive ACS model taking into account ICP, HD, LoS, EHR as well as demographic and geographic variables remains to be described.

Using descriptive, comparative, and spatial analysis tools as well as cartographic representation, we assessed inter-regional disparities in the management of ACS. More specifically, our main objectives were to describe and compare the regional rates of ICP, HD, EHR, and also the average LoS after an ACS in 2000 in the province of Quebec. We also assessed whether there is a relationship between ICP and HD, LoS, and EHR. Secondary objectives intended to determine if the proximity of a specialized cardiology center influences the ICP, HD, LoS and EHR. Finally, we assessed the extent to which the relationships between these variables vary spatially.

## Methods

### Design

We conducted a population-based cohort study using data from the Quebec's hospital discharge register. This register provides administrative data on patients hospitalized in acute care hospitals in the province of Quebec. Studies confirming the validity of the administrative hospital discharge data concerning myocardial infarction have previously been published [12,13].

### Studied population

For the studies on ICP, HD, and LoS, the studied population consisted of all patients 25 years and older living in the province of Quebec, who have been hospitalized in Quebec for ACS between January 1^st ^and December 31^st ^2000. For the study on EHR, we have selected those patients who survived the index hospitalization. The "index hospitalization" was the first hospitalization during the study period. We included patients who were hospitalized for acute myocardial infarction (code 410 of the International Disease Classification, 9^th ^revision (IDC-9)) or other acute or subacute forms of ischemic cardiopathy (IDC-9 code 411) as the main diagnosis. Patients with an unknown geographic location code were excluded.

### Data sources

Attributive and spatial data were used. Attributive data included all patient-data. This was obtained from the Quebec's hospital discharge register and the death register. Each patient was spatially referenced by his/her postal code of residence using data from ESRI [14], DMTI Spatial [15] and from the Quebec Ministry of Health and Social Services [16,17]. The geographic coordinate system used for the cartographic presentation was GCS North American 1983.

### Studied variables

Patients were considered to have undergone an invasive cardiac procedure (ICP) if there was mention of an angiography, angioplasty or aortocoronary bypass as coded in the Quebec's hospital discharge register (Canadian Acts Codes beginning with 480 to 483 or 489) for the index hospitalization. The hospital death (HD) was defined as in-hospital death at the index hospitalization. The length of stay (LoS) was defined as the number of days between the patient's admission and discharge from the index hospitalization. If the care for ACS was delivered over several contiguous hospitalizations involving hospital transfer, the presence of an ICP, HD and the LoS were evaluated for the entire episode. The early hospital readmission (EHR) was defined as a hospital readmission for heart disease as the main diagnosis (ICD-9: 410 to 414) in the first 30 days following discharge from the index hospitalization. Distance to a specialized cardiology center, geographic location and the patient's age and gender were used as covariates. Specialized cardiology centers are hospitals that provide technical facilities and professional expertise in cardiology. These centers are the only hospitals that provide invasive cardiac procedures. The 16 specialized centers were identified via the *Quebec tertiary cardiology network*. The aerial distance to a tertiary cardiology center was categorized into a variable taking the value 1 if the residence was within an aerial distance of 32 km from the nearest specialized cardiology center (as defined by the geometrical centroid of the postal code area), the value 2 if the residence was between 32 and 64 km from the nearest cardiology center, the value 3 if the residence was between 64 and 105 km, and finally, the value 4 if the residence was farther than 105 km from the nearest cardiology center. We chose these cut points based on transportation time of respectively 60, 90 and 120 minutes to cover a distance of 32, 64 and 105 km [11,18]. The localization of the residence was defined by the geometrical centroid of its postal code. The geographic grouping was based on the health administrative region of the patient's home location. Because of their geographic similarities and their small populations, the *Nunavik *and *James Bay Cree Lands *were merged with the Northern Quebec region.

### Statistical analyses

Descriptive analyses by age, gender and place of residence (administrative region) were done. Incidences of hospitalization for ACS were calculated in regard to the estimated population for 2000 [19]. We used the Pearson χ^2 ^test for comparisons between proportions and the Fisher F-statistics for comparisons of means (ANOVA after a logarithmic transformation) in the studied groups (age, gender, and region) [20]. For the 16 Quebec administrative regions, we calculated the standardized ICP, HD, and EHR ratios [21], as the ratios between observed and expected numbers given age and gender. We also calculated the standardized LoS as the age and gender weighted average of the LoS.

**Figure 1:**
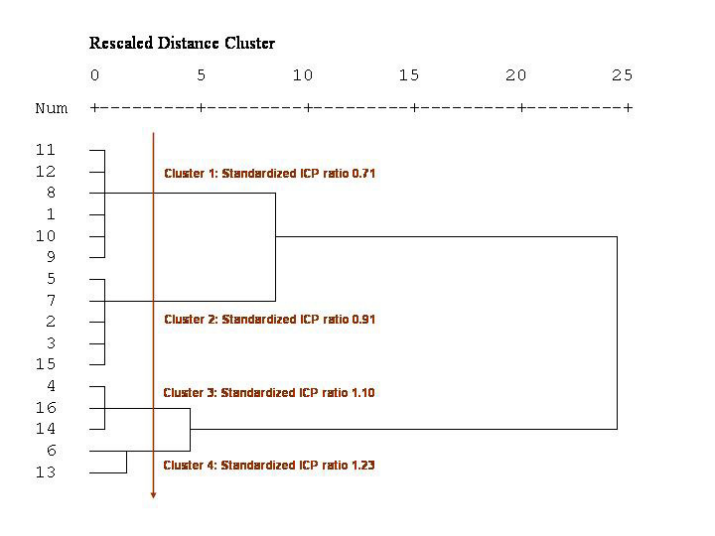
Dendrogram produced by a hierarchical cluster analysis of the standardized ICP ratios.

Using a Hierarchical Cluster Analysis [22], we grouped in different classes the standardized ICP, HD, and EHR ratios as well as the LoS weighted average for the 16 regions. Hierarchical clusters analysis (HCA) is based on measures of similarities (defined by the squared Euclidean distance between the values) computed from values of one or several variables; at a first step, each case forms a cluster, then the two nearest or similar clusters are grouped to form a new cluster, and so on, until an appropriate number of clusters is reached or until all cases are grouped into a unique cluster. The *centroid clustering *method was used. The choice of the number of clusters was based on visual inspection of the dendrograms produced by the method, the idea being to display a sufficient number of clusters in order to have homogeneous groups (within-group homogeneity) but dissimilar enough (inter-group heterogeneity) (see Figure 1 for an example of dendrogram). In most of the methods of classification available in ArcGIS [23] (equally spaced, quintiles, natural breaks, mean and standard deviation method, etc), the number of groups must be *a priori *fixed, and the choice of the method often depends on the distribution of the data. We chose to use a HCA instead of these usual grouping methods because HCA can be used regardless of the data distribution and because we can choose the appropriate number of groups after a visual inspection of the dendrogram.

Multiple linear (log-normal) regression analysis was performed on LoS and multiple logistic regression analyses were performed on ICP, HD and EHR. Beyond age and gender, the potential predictors of the average log-transformation of LoS, the HD rate and the EHR rate were the presence of an ICP and the distance to a specialized cardiology center. All main effects were included in the models, but we allowed some interaction terms, *gender *× *ICP*, *distance *× *ICP*, and *Gender *× *distance*, to enter the models only if they were statistically significant at a 0.05 level. The parameter estimates between outcomes and predictors together with their 95% confidence intervals were calculated.

The residuals derived from the global models were also calculated at a regional level and mapped. In the case of the log-normal model of LoS, we used the usual residuals measured as the difference between the observed mean and the mean of the expected (after a log-transformation of LoS). The residuals used in the logistic models for ICP, HD and EHR were, at a regional level, the signed deviance residuals, given by:



where *y *is the number of observed event (ICP, HD or EHR) in the region and *μ *is the expected number of event (ICP, HD or EHR) in the region given by the global model.

To see whether or not the association between outcomes and the potential predictors varied spatially (constant parameters or spatially varying parameters), we used a *Geographically Weighted Regression *(GWR) approach proposed by Fotheringham et al [24] on a random sample of 20% of the total cohort. A random sample was necessary because of limitations in the execution time of the GWR software. The GWR approach extends the traditional global regression framework



where regression parameters are constant over the whole study region, by allowing local rather than global parameters to be estimated so that the model is rewritten as:



where (*u*_*i*_,*v*_*i*_) denotes the coordinates in the *i*th point in space and the parameters now vary over the study region with geographic coordinates (spatial variability in parameters). In the case of the logistic regression, the left side of the equations above (*y*_*i*_) is replaced by logit [Prob(*Y*_*i *_= 1|*x*_*i*_], where logit (*p*) = log[*p*/(1-*p*)]. The difference between traditional regression and GWR is that, as opposed to traditional regression, GWR assumes implicitly that observed data near to location *i *have more of an influence in the estimation of the local parameters than do data located farther from *i*. In essence, the GWR model measures the relationships inherent in the model around each location *i*. In the case of the log-transformation of LoS, a Monte Carlo significance test was used to infer on the spatial variability of the parameters, whereas in the logistic models, because of the unavailability of this test in the GWR software [25] for binary variables, we got a "feel" for the degree of variability in the regression coefficients by comparing the inter-quartile range of the local estimates with the standard error of the global estimate; a range between the upper and the lower quartiles (corresponding to 50% of all local estimates) much higher than two times the standard error (corresponding to 68% of the area of a normal distribution) indicates a spatial variability in the relationship [25]. The global regression analyses and residuals analyses were done using SAS Release 8.02 [26], the hierarchical cluster analyses were done using SPSS Release 11.0.1 [27] and the local estimates of the relationships between outcomes and predictors were estimated using GWR Release 3 [25]. Cartographic representations were done using ArcGIS Release 8.3 [23].

### Ethical considerations

This project was approved by the Sherbrooke University Hospital Ethics Board and the *Commission d'accès à l'information du Québec *(Quebec Commission to Information Access).

## Results

A total of 24,564 patients have been hospitalized for ACS in Quebec between January 1^st^, 2000 and December 31^st^, 2000. Of those, 20 patients were excluded because they were less than 25 years old or because there was an error in their administrative code of residence. Therefore, the study population totalled 24,544 patients, men accounting for 63% (*n *= 15,481) of it. The average age of this population was 66.7 years (± 13.0).

The incidence of hospitalization for ACS varied greatly according to gender and age with the highest rates observed in men and very old people (Table 1). A total of 1699 (6.9 %) individuals died during the index hospitalization and the HD rates varied according to gender and age (*p *< 0.0001). An ICP rate in Quebec of 43.7% was much lower for women than for men (*p *< 0.0001), and decreased with age, whereas the hospital LoS showed an opposite trend. Also, neither gender nor age seemed to influence the EHR rate. Finally, regional heterogeneity was observed in all outcomes considered in this study, namely, in incidence rates (range: 340 – 827 per 100,000 inhabitants; p < 0.0001), in ICP rates (range: 29.4 – 51.6%; p < 0.0001), in HD rates (range: 3.3 – 8.2%; p < 0.0001), in average LoS (range: 7.5 – 14.4 days; p < 0.0001), in median LoS (range: 5 – 10 days), and in EHR rates (range: 4.7 – 14.2%; p < 0.0001).

**Table 1:** Acute coronary syndrome (ACS) in Quebec in 2000, ACS hospitalization incidence (INC), invasive cardiac procedure (ICP) rate, hospital death (HD) rate, average (median) length of stay (LoS) (days), and early hospital readmission (EHR) rate by gender, age and region

	*Number*	INC*§>	*ICP *^§ ^*n (%)*	*HD *^§ ^*n (%)*	*LoS *^§ ^*Mean (Median)*	EHR**n (%)
*TOTAL*	24,544	484	10,725 (43.7)	1699 (6.9)	11.5 (8)	1893 (8.3)

*Women*	9063	347	3312 (36.5)	829 (9.2)	12.3 (8)	650 (7.9)
*Men*	15,481	629	7413 (47.9)	870 (5.6)	11.0 (7)	1243 (8.5)

*Age < 55 yrs*	4955	146	2687 (54.2)	69 (1.4)	8.7 (6)	406 (8.3)
*Age 55–64 yrs*	5315	721	2886 (54.3)	152 (2.9)	10.6 (7)	424 (8.2)
*Age 65–74 yrs*	6678	1234	3329 (49.8)	389 (5.8)	12.7 (8)	504 (8.0)
*Age 75–84 yrs*	5652	1833	1697 (30.0)	684 (12.1)	13.2 (8)	427 (8.6)
*Age ≥ 85 yrs*	1944	2046	126 (6.5)	405 (20.8)	11.9 (8)	132 (8.6)

*1 Lower St. Lawrence*	801	566	260 (32.5)	57 (7.1)	11.7 (8)	61 (8.2)
*2 Saguenay-Lac-St-Jean*	835	438	361 (43.2)	50 (6.0)	8.7 (6)	74 (9.4)
*3 Quebec City*	2278	493	925 (40.6)	155 (6.8)	12.5 (8)	145 (6.8)
*4 Mauricie, Central Qc*	1870	558	860 (46.0)	153 (8.2)	14.4 (10)	136 (7.9)
*5 Eastern Townships*	1269	645	468 (36.9)	73 (5.8)	11.3 (8)	125 (10.4)
*6 Montreal-Center*	5282	406	2728 (51.6)	428 (8.1)	11.7 (7)	353 (7.3)
*7 Outaouais*	733	340	294 (40.1)	56 (7.6)	7.5 (5)	32 (4.7)
*8 Abitibi-Témiscamingue*	614	615	195 (31.8)	31 (5.0)	10.1 (7)	57 (9.8)
*9 North Shore*	449	664	154 (34.3)	15 (3.3)	11.8 (7)	60 (13.8)
*10 Northern Quebec*	93	447	36 (38.7)	4 (4.3)	9.8 (6)	9 (9.8)
*11 Gaspé, Magdalen Is*.	590	827	177 (30.0)	20 (3.4)	11.5 (8)	56 (9.8)
*12 Chaudière-Appalaches*	1530	583	449 (29.4)	106 (6.9)	10.2 (7)	202 (14.2)
*13 Laval*	1153	477	595 (51.6)	78 (6.8)	11.7 (7)	71 (7.4)
*14 Lanaudière*	1417	534	716 (50.5)	82 (5.8)	10.2 (7)	99 (7.4)
*15 Laurentians*	1611	509	667 (41.4)	88 (5.5)	10.5 (8)	126 (8.3)
*16 Montérégie*	4019	453	1840 (45.8)	303 (7.5)	12.0 (8)	287 (7.7)

* Per 100,000 inhabitants

^§ ^The difference between gender, age and regions is statistically significant (p < 0.0001)

** Early hospital readmission rates are calculated for ACS survivors only. The difference between gender, age is not statistically significant (p = 0.1065; 0.8357) but significant between regions (p < 0.0001)

**Figure 2:**
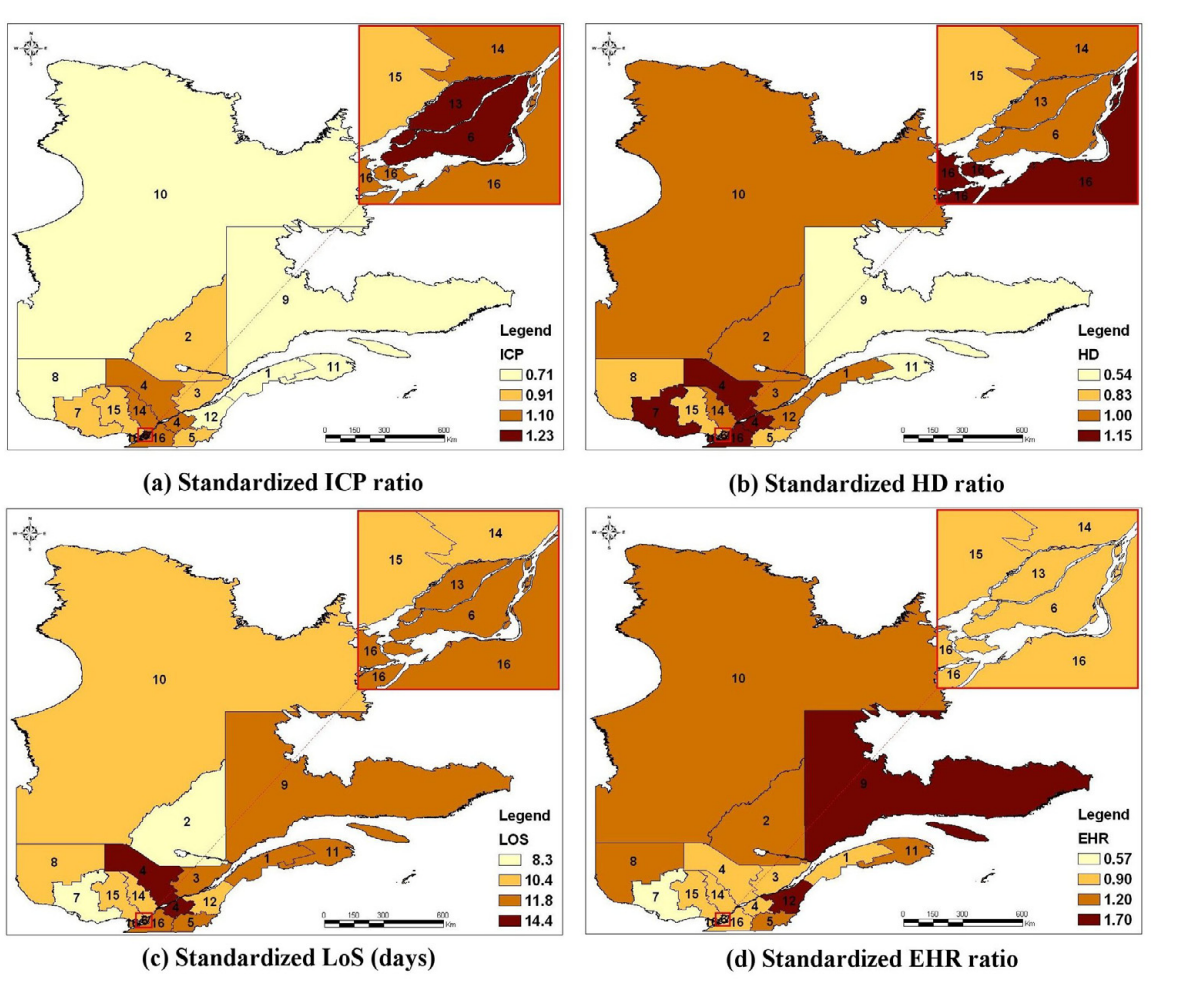
Maps of the standardized ratios* for invasive cardiac procedures (ICP), hospital death (HD), early hospital readmission (EHR), and of the standardized length of stay (LoS) after classification** ^§^. * Standardized for age and gender **Administrative regions were grouped in 4 homogeneous groups (hierarchical cluster analyses). The number beside each colour represents the overall standardized ratio (or the average) of the associated group ^§ ^Administrative regions: 1 : Lower St. Lawrence; 2 : Saguenay-Lac-St-Jean; 3: Quebec City; 4: Mauricie, Central Quebec; 5: Eastern Townships; 6: Montreal Center; 7: Outaouais; 8: Abitibi-Témiscamingue; 9: North Shore; 10: Northern Quebec; 11: Gaspé, Magdalen Islands; 12: Chaudière-Appalaches; 13: Laval; 14: Lanaudière; 15: Laurentians; 16: Montérégie

The map of the age/gender standardized ICP ratio (Figure 2a) highlights a decreasing gradient from Montreal metropolitan areas to peripheral regions. The cartographic representation of the age/gender standardized HD ratio (Figure 2b) shows that very low HD rates are observed in some remote regions. The trend in the age/gender standardized LoS (Figure 2c) shows patches of high average levels around South Center and very low levels at South Western. Finally, the cartographic representation of regional age/gender standardized EHR ratio (Figure 2d) shows an increasing gradient from peripheral regions to Montreal metropolitan areas.

Multiple regression analyses (Table 2) show that the presence of an ICP is correlated with an increased LoS, but with a decreased HD and EHR rates. Furthermore, patients living within 32 km to a specialized cardiology center had a higher likelihood of having an ICP at the index hospitalization, a shorter LoS, and a lesser likelihood of being readmitted within 30 days. The association between distance and HD is not statistically significant. Also, women received less ICP, stayed longer at index hospital, but had less early hospital readmissions. As shown in the interaction term between ICP and distance to a specialized cardiology center, we observe that the LoS is higher for patients with an ICP, but no clear trend is shown according to distance. On the other hand, it is clearly shown that for those patients with ICP, the closer they are from the nearest cardiology center, the lesser they stay at the hospital.

**Table 2:** Results from multiple logistic regression models for Invasive cardiac procedure (ICP), hospital death (HD), and early hospital readmission (EHR), and from the log-normal regression model for the length of stay (LoS)^§^

	*ICP β parameter (95% CI)*	*HD β parameter (95% CI)*	*LoS β parameter (95% CI)*	*EHR β parameter (95% CI)*
Intercept	2.600 (2.455;2.744)	- 7.004 (-7.393; -6.616)	1.006 (0.937;1.075)	- 1.926 (-2.190;-1.662)

**Main effects**				

*Age*	- 0.040 (-0.042;-0.037)	0.066 (0.061;0.071)	0.011 (0.010;0.012)	- 0.003 (-0.007;0.001)

*Women vs Men*	- 0.200 (-0.257;-0.144)	- 0.103 (-0.222;0.015)	0.039 (0.013;0.064)	- 0.237 (-0.360;-0.113)

*ICP*	-	- 0.980 (-1.152;-0.807)	0.470 (0.439;0.501)	- 0.838 (-0.964;-0.711)

*Distance cardiology Center*				
*32–64 km vs <32 km*	- 0.247 (-0.323;-0.170)	- 0.132 (-0.285;0.022)	0.003 (-0.042;0.048)	0.138 (0.003;0.274)
*64–105 km vs <32 km*	- 0.509 (-0.603;-0.416)	0.106 (-0.062;0.273)	- 0.083 (-0.135;-0.032)	0.356 (0.207;0.505)
*≥105 km vs <32 km*	- 0.486 (-0.563;-0.409)	- 0.148 (-0.301;0.004)	0.040 (-0.003;0.083)	0.128 (-0.005;0.262)

**Interaction terms**				

*Women *× *ICP*	-	0.4327 (0.181;0.685)	-	0.367 (0.151;0.583)

*ICP *× *Distance*	-	-		-
*32–64 km vs <32 km*			0.138 (0.069;0.207)	
*64–105 km vs <32 km*			0.276 (0.192;0.360)	
*≥105 km vs <32 km*			0.350 (0.280;0.419)	

^§ ^CI: Confidence interval; A positive parameter estimate indicates an increased risk whereas a negative estimate indicates a reduced risk

**Figure 3:**
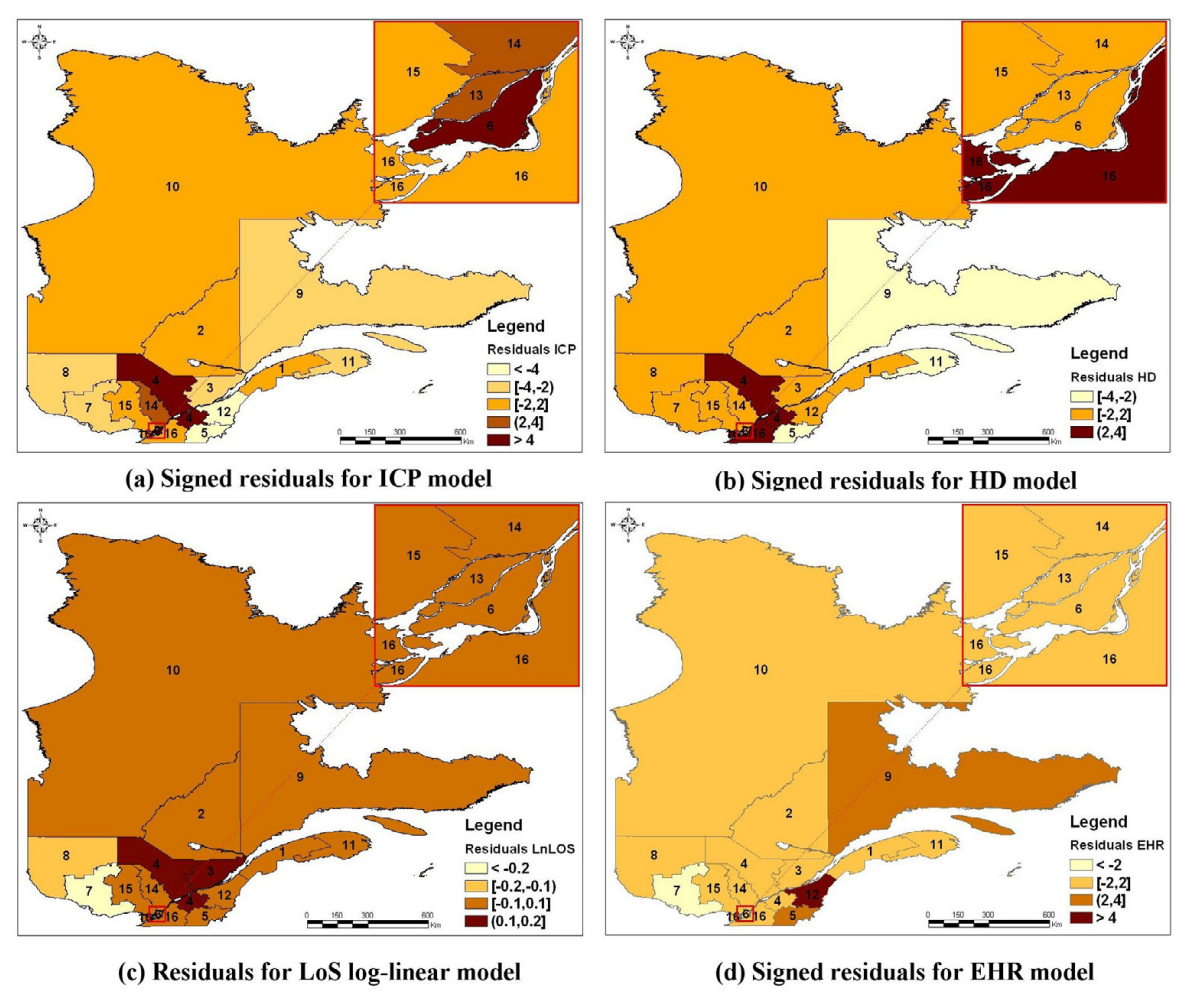
Maps of the residuals derived from the multivariate log-linear model for the length of stay (LoS) and the signed residuals* derived from the multivariate logistic model for the invasive cardiac procedure (ICP), the hospital death (HD), and the early hospital readmission (EHR) **^§^. * The signed residuals are defined as:  **Administrative regions were grouped in 4 homogeneous groups (hierarchical cluster analyses). The number beside each colour represents the overall standardized ratio (or the average) of the associated group ^§ ^Administrative regions: 1 : Lower St. Lawrence; 2 : Saguenay-Lac-St-Jean; 3: Quebec City; 4: Mauricie, Central Quebec; 5: Eastern Townships; 6: Montreal Center; 7: Outaouais; 8: Abitibi-Témiscamingue; 9: North Shore; 10: Northern Quebec; 11: Gaspé, Magdalen Islands; 12: Chaudière-Appalaches; 13: Laval; 14: Lanaudière; 15: Laurentians; 16: Montérégie

A cartographic representation of the residuals associated to each global model is presented in Figure 3. These maps show that the heterogeneity observed between regions in the ICP, HD, and EHR rates, as well as in the log-transformation of the LoS, cannot be explained totally by age, gender, ICP (when applicable), and distance to a specialized cardiology center.

**Figure 4:**
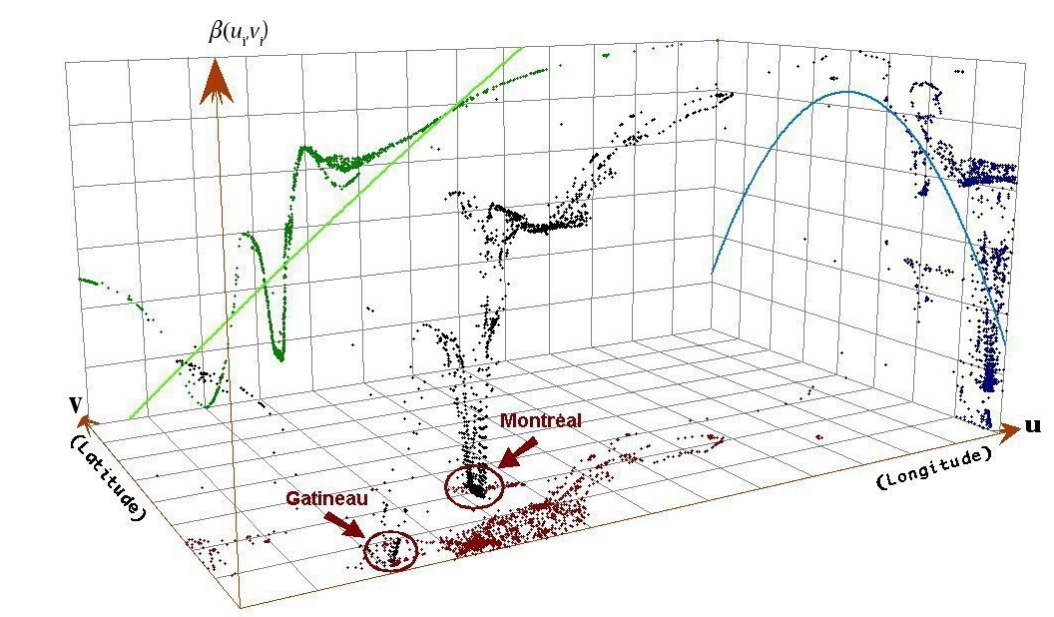
Trend analyses of the local relationships *β*(*u*_i_, *v*_*i*_) between ICP and the log-transformation of the length of stay (LoS).

Some of the relationships *β*(*u*_*i*_, *v*_*i*_) between covariables and LoS are not uniform over the study surface. Indeed, when considering the log-normal model for LoS, the Monte Carlo significance test shows significant spatial variations in the GWR local estimates of the parameters associated to the covariables ICP (median estimate: 0.55, min: 0.26, lower quartile: 0.35, upper quartile: 0.73, max: 0.95, *p *< 0.0001) and distance to a cardiology center (median estimate: 0.0015, min: -0.0014, lower quartile: 0.0004, upper quartile: 0.0031, max: 0.0107, *p *< 0.0001), whereas it shows no significant spatial variability in the local estimates of the parameters associated to gender (*p *= 0.60) and age (*p *= 0.86), that is, in favor of constant regression parameters. Moreover, trend analyses of the parameter estimates show that an increased relationship between ICP and LoS is observed as we move away from *Montreal *and the city of *Gatineau *in *Outaouais *(Figure 4), but no clear trend is observed in the local relationships between distance to a specialized cardiology center and LoS. The local estimates of the relationship between ICP and HD varied from -0.94 to -0.58 with a median estimate of -0.84 (lower quartile: -0.84, upper quartile: -0.78), corresponding to a variation in the odds ratios from 0.39 to 0.56. Also the local estimates of the relationship between ICP and EHR varied from -0.79 to -0.36 with a median estimate of -0.39 (lower quartile: -0.48, upper quartile: -0.38). Nevertheless, no clear spatial variation was suspected in the GWR local estimates of the parameters associated to the covariables in the multiple logistic regressions for HD and EHR (Coefficients of variation: 7.9% and 12.5% respectively).

## Discussion

Regional heterogeneity was observed in all outcomes. These spatial heterogeneities are not surprising since we are dealing with multivariate health processes evolving across social, economic and environmental explanatory related variables for which local conditions (physical environment and human activity) are very important [28].

Our results show an overall ICP rate of 43.7% in Quebec for the year 2000. This rate is higher than what has been reported in a previous study conducted by the CCORT group [29] in which the 30-day revascularization (angioplasty or aortocoronary bypass) rate in Quebec for the fiscal year 1999/2000 was 26%. This difference is probably due to the fact that, contrary to the CCORT study, we added the angiography without revascularization procedure in the definition of ICP. We did find relevant to include it in the definition of ICP as both this diagnostic procedure and revascularization are available only in a specialized cardiology center. By limiting our definition of ICP to revascularization procedures only, our observed provincial rate would be down at 34%. The remaining 8% difference between revascularization rates of 34% and 26% can be explained by the fact that the data used in this study is nearly one-year more recent than the data used by the CCORT group. One can also consider the fact that revascularization is more and more implemented in practice as a first intent procedure.

We obtained an average LoS of 11.5 days (median LoS: 8 days), this is slightly higher than what other Canadian researchers have observed. Hall and Tu [6] have reported an average LoS for myocardial infarction in Quebec for the fiscal year 1999/2000 of 9.7 days after adjustment for age, gender and ICP. This difference is likely attributable to a difference in methodology as Hall and Tu excluded from their analysis patients with a LoS beyond the 97.5 percentile.

We also observed gender variations in ICP rates, HD rates and LoS. Lower ICP rates and a longer LoS for older women have also been reported by other authors [30-33]. It has been argued that this difference between genders could however reflect a difference in the treatment indications rather than a difference based on gender [34].

After discharge from the index hospitalization, 8.3% of the population alive was readmitted within 30 days with a diagnosis of coronary heart disease. Some regions display extreme and opposite results. On one hand, the extremely low rate of EHR observed in the Outaouais region probably reflects an underestimation due to the close proximity to a specialized cardiology center located in the adjacent province. In fact, the geographical proximity of the Outaouais city of Gatineau located in the province of Quebec and the city of Ottawa located in the province of Ontario makes it difficult to interpret the data from this region. On the other hand, the high EHR rate observed in the *Chaudière-Appalaches *and the *North Shore *regions cannot be explained with variables used in the study. Differences observed in early hospital readmission rates probably reflect more the difference in the managed cares than in health outcomes like morbidity. In fact, elevated rates in some regions may have been due to discharge after stabilization and then elective readmission at a specialized cardiology center for an ICP.

An important finding of this study is the inverse relationship between ICP and HD, the inverse relationship between ICP and EHR, the increase of LoS with ICP, the positive relationship between the distance to a specialized cardiology center and EHR as well as LoS, and the negative relationship between the distance to a specialized cardiology center and ICP. The paradoxical finding of decreased LoS with proximity to specialized cardiology center while we find an increased LoS with ICP and an increased likelihood of ICP with proximity can be explained by the interaction between ICP and proximity to cardiology center in the evaluation of LoS. As seen in Table 2, the interaction term included in the LoS model show that, for patients with ICP, the LoS is lower for patients near a cardiology center than for those farther, whereas, for patients not receiving ICP, those that are closer have not a lower LoS than the others. We can also argue that patients living far from a specialized cardiology center will stay longer at the hospital during the whole episode of care if they received an ICP because of hospital transfer from non-specialized to a specialized cardiology center. An explanation for the lack of association between distance and HD might be survival bias, that is, individuals in rural areas with ACS may be less likely to survive to hospitalization. Sicker patients in urban areas would be able to make it to the hospital.

Moreover, the regional variability of the relationships between ICP and the distance to a specialized cardiology center with regards to the LoS is another interesting result. Accessibility to ICP facilities is a potential explanatory factor involved in the utilization of the ICP regularly put forward in the literature [35]. In our study, as shown by the maps of the residuals, variability in HD, LoS, and EHR can only be partly explained using a model taking into account ICP, accessibility to a specialized cardiology center, age and gender alone.

A study by Scott [11] on the accessibility of specialized treatments for vascular cerebral accidents reports that the proximity of a specialized care center favours young and rich populations at the expense of older, native and underprivileged populations. According to Alter [36], geographic factors and accessibility to services do not explain the gradient of angiography use after a myocardial infarction in Ontario, and the author suggests that the observed regional differences in revascularization rates might actually reflect differences in regional socioeconomic factors such as age and socioeconomic status. However, these authors used a distance threshold of 50 km instead of 32 km which may reduce the strength of the link between distance and the use of specialized cardiology services.

There is a need to better define, taking adjustment variables into account, what the ideal thresholds should be to appropriately describe access to ICP.

To better understand geographical disparities in cares and health outcomes of ACS, there is a need to explore more comprehensively the contribution of socio-demographic variables. Rurality is one of these variables that are worth the effort to consider in subsequent studies [37]. Indeed, rural populations differ from urban ones not only because of the distance between them but also because they share different cultural and socioeconomic backgrounds. Among other variables that could also be important are social and material deprivation indices [38], in addition to medical care variables and professional attributes including academic affiliation and year of graduation for family physicians and cardiologists. Finally, one should also consider other major determinants of ACS outcomes, namely concomitant diseases like diabetes, hypertension, congestive heart failure, etc., and the use of secondary preventive drugs like angiotensin converting enzyme inhibitors, β-blockers, statins, and platelets inhibitors [39].

This study has some limitations. The nature of administrative data used in this study did not allow discriminating between planned readmission and readmission for a distinct ACS event. Even though the Quebec's hospital discharge register has been used for acute myocardial infarction [12,13], the follow-up of the hospital's care episode spread out over different care institutions requires the construction of an algorithm, the accuracy of which needs to be further validated.

Even if we observed that ICP increased the LoS but reduced the HD and EHR, we cannot argue that ICP increases the quality of care. In fact, recent work suggests that EHR might be an indicator of good medical care. However, it has been suggested that there is a complex network of factors influencing medical care, EHR and the association between them [40]. Linking the EHR measure to good medical care is still controversial. While some analyses [41] showed that the EHR rate was higher when care was less appropriate, other authors [42] argued that there was no statistically significant association and suggested that the slight association might reflect the difficulty in measuring quality of care. Moreover, how the hospital LoS relates to quality of care is controversial, this association seeming to vary according to how quality of care is defined. Some authors have observed a positive association between LoS, treatment and discharge scores [43] ; others have observed the opposite when defining quality of care based on physician judgments [44], whereas others have reported no significant relationship between LoS and quality of care as defined by readmission or mortality rates [45,46]. In this study, since the hospital readmission could have been planned, we definitely cannot use the early hospital readmission as an indicator of good medical care.

This study aimed at understanding medical care for ACS in order to model important administrative and health related outcomes. To our knowledge, this is the first to use advanced spatial statistical analysis with medico-administrative data from an entire province in Canada to build a model that could be later refined with the inclusion of other meaningful individual, socio-demographic and health care variables. Regional disparities in the province of Quebec, as highlighted by this study, may well represent an adaptation of the health care system for geographical disparities in order to deliver good quality of care, despite major limitations in terms of physical accessibility to specialized cardiology centers. To test this hypothesis, it would require a very complex research design, using qualitative and quantitative analyses. Such studies would have to take into account determinants of care at many levels such as: at the sociological, cultural, political, and economical level, as well as at the professional and geographical level [47,48]. In terms of policy impact, it could also mean that regional based decision making may provide valuable contribution in the management of care for ACS, taking into account the limited physical accessibility of specialized cardiology centers.

## Conclusion

An important finding of this study is the inverse relationship between ICP and HD, the inverse relationship between ICP and EHR, the increase of LoS with ICP, the positive relationship between the distance to a specialized cardiology center and EHR as well as LoS, and the negative relationship between the distance to a specialized cardiology center and ICP. Moreover, the regional variability of the relationships between ICP and the distance to a specialized cardiology center with regards to the LoS is another interesting result. The EHR rates are clearly related to ICP and geographic patterns observed could reflect to some extent patients' accessibility to revascularization specialized settings. Further studies are needed to clarify the nature of the link between geographical influence, ICP and EHR.

## Competing interests

This project has benefit from an unrestricted grant by Merck Frosst Canada Ltd. J.-P. Grégoire was employed by Merck Frosst Canada Ltd.

## Authors' contributions

AV, TN and JPG conceived the study, GB and AH participated to the geographical aspect of the study, JL performed the literature review on acute coronary syndrome, JC and TN performed the analyses. AV, TN, JPG and JC participated to the writing of the manuscript. AH produced the maps.

## Pre-publication history

The pre-publication history for this paper can be accessed here:


